# Changing the Ethics of the Medical Profession

**Published:** 1873-02

**Authors:** 


### Editorial.

### Changing the Ethics of the Medical Profession.

The Oneida County Medical Society sends us Circular, with resolutions proposing to the profession to offer consultation with all *legally* qualified physicians. The proposition is too absurd for discussion, and when considered in practical operation its impossibility is sufficiently apparent. Do Homœopathic or Eclectic physicians want our advice: generally, not much. Do regular physicians want their advice; do they respect their opinions; can they follow their example or teaching; do they in any case propose to adopt their practice? The Governor should be asked to appoint a commission of experts on insanity to inquire into the mental equilibrium of Oneida County doctors, the resolutions which they passed and circulated throughout the State indicating plainly that they are not "level." Legal qualification to practice medicine and surgery in the State of New York, does not mean anything at all; every man, woman and child in the State has the legal right to practice medicine and surgery. The possession of a case of unmedicated sugar pellets, or some tinctures of indigenous plants with accompanying pretentions, entitle to membership in the county societies; and the idea of meeting in consultation, those who have assumed the title of doctor and practice under it, is the most inconsistent proposition conceivable. But looking at the central idea of the society, we would only make three inquiries. Do physicians desire the advice of their irregular neighbors; or do their neighbors feel the need of, and desire advice from, regular physicians? Do the public care to join regular medicine with the various systems and ists and pathies they have chosen, inherited, or fallen into? These questions being answered and we think the whole field is sufficiently canvassed. If the Mahometan does not believe that his religion is as good as any, and applicable in all casas to the salvation of his soul, let him reject it and act accordingly.

